# Healthcare-seeking with bothersome lower urinary tract symptoms among men in the Danish population: the impact of lifestyle and socioeconomic status

**DOI:** 10.1080/02813432.2019.1608412

**Published:** 2019-05-06

**Authors:** Ann Rubach, Kirubakaran Balasubramaniam, Maria Munch Storsveen, Sandra Elnegaard, Dorte Ejg Jarbøl

**Affiliations:** Research Unit of General Practice, Department of Public Health, University of Southern Denmark, Odense C, Denmark

**Keywords:** Lower urinary tract symptoms, Men, General practice, Socioeconomic status, Lifestyle, Health behavior

## Abstract

**Objective:** (1) To identify possible factors of importance for reporting lower urinary tract symptoms (LUTS) among men and (2) to examine possible associations between socioeconomic status (SES), lifestyle factors, and likelihood of men contacting a general pracitioner (GP) regarding LUTS reported to be of concern or influencing daily activities (bothersome LUTS).

**Design:** Nationwide population-based, cross-sectional survey. Data was collected in 2012.

**Setting:** The general Danish population.

**Subjects:** A total of 48,910 randomly selected men aged 20+.

**Main Outcome Measures:** (1) Odds ratios for reporting LUTS by lifestyle and SES, and (2) Odds ratios for GP contact with bothersome LUTS by lifestyle and SES.

**Results:** 23,240 men participated (49.8%). Nocturia was the most commonly experienced LUTS (49.8%). Incontinence was most often reported as bothersome (64.1%) and nocturia less often reported as bothersome (34.2%). Only about one third of the men reporting a bothersome LUTS contacted their GP. Odds for reporting LUTS significantly increased with increasing age, obesity, and lack of labor market affiliation. Increasing age and symptom burden significantly increased the odds for GP contact regarding bothersome LUTS. No overall associations were found between lifestyle, SES, and GP contact.

**Conclusion:** Bothersome LUTS are common among Danish men. Concern and influence of LUTS on daily activities are important determinants of GP contact, yet only one in three bothersome LUTS are discussed with a GP. Advanced age and symptom burden were significantly associated with GP contact.

**Implications:** Information on treatment options for LUTS might be desirable among Danish men regardless of SES and lifestyle.

**Key points**
- Urological symptoms are common among men in the Danish population and are often managed without contacting healthcare professionals.
- Increasing age and symptom burden significantly increase the likelihood of consulting a general practitioner regarding bothersome urological symptoms
- Healthcare-seeking behavior with bothersome urological symptoms is not influenced by lifestyle or socioeconomic status among Danish men;
- Information about available, effective treatment options for urological symptoms might be desirable among men regardless of socioeconomic status and lifestyle

## Introduction

Lower urinary tract symptoms (LUTS) are reported commonly among men worldwide and the prevalence increases with age [1]. LUTS include various urological symptoms and can be divided into three different subgroups; storage, voiding, and post micturition symptoms [2]. The prevalence of LUTS varies considerably in different studies, with estimates ranging from 39–90% [1,3,4], primarily due to differences in study design and definitions of LUTS.

Several conditions can cause LUTS, including bladder overactivity, weakness of the detrusor muscle, urinary tract infection, and prostate cancer, but the most common cause is benign prostate hyperplasia (BPH), which is age-related enlargement of the prostate [2]. If BPH is left untreated, the prostate may continue to increase in size as BPH is a progressive condition, thus leading to acute urinary retention that requires immediate medical attention [5].

Besides age and BPH, other factors seem to increase the risk of experiencing LUTS e.g. lifestyle factors such as obesity [6], alcohol intake, and smoking [7]. A high educational level has been associated with a decreased likelihood of LUTS [7]. These studies are carried out in small selected samples and results remain to be confirmed in larger study populations.

Though LUTS are often caused by benign medical conditions, the experience of LUTS is shown to have a negative impact on quality of life and is associated with embarrassment, shame, depression, and anxiety [8–10]. Although several treatments are effective in reducing urological symptoms [11], the majority of men experiencing LUTS manages their symptoms privately, without medical help [3,12].

Increasing age, severity of symptom(s), concern regarding the symptom(s), and magnitude of influence on daily activities are all positively associated with healthcare-seeking among men with urological symptoms [3,12]. However, only half of the LUTS reported to be of extreme concern or influence on daily activity were discussed with a GP in a previous study [3].

The literature indicates that socioeconomic status (SES) and lifestyle factors influence the decision to contact a GP when experiencing different symptoms [13–17]. This has, however, not yet been examined specifically among men with LUTS reported to be of concern or with influence on daily activities.

The objectives of this study were therefore to (1) identify possible factors of importance for reporting LUTS among men and (2) examine possible associations between SES, lifestyle factors and likelihood of men contacting a GP with LUTS reported to be of concern or with influence on daily activity.

## Material and methods

### Study design and population

This population-based, cross-sectional study is a part of the Danish Symptom Cohort. The study was designed as a nationwide cohort study of 100.000 adults aged 20 years or older, randomly selected from the general population through the Danish Civil Registration System (CRS) and invited by postal letter to participate in an online survey [18]. Two weeks later, a reminder letter was sent to non-respondents. After a further two weeks, non-respondents were contacted by telephone to encourage them to participate. Respondents lacking internet access were offered the option of participating in the survey by telephone interview.

### Questionnaire

The methodological framework for developing the survey has been thoroughly described by Rasmussen et al. [18]. A comprehensive questionnaire containing 44 predefined symptoms was designed. Six urological symptoms, all known to commonly occur in the general population and assumed to be primarily of benign medical cause, form the basis of this paper [1–3]. Five were storage symptoms (frequent urination, nocturia, urge incontinence, stress incontinence, and incontinence without stress/urge) and one was a voiding symptom (difficulty in emptying the bladder).

The survey asked the participants if they had experienced any of the six symptoms in the preceding four weeks and, if so to what degree the symptom had been concerning or had influenced their daily activities. A five-point Likert scale was used for response options: *not at all, slightly, moderate, quite a bit* and *extremely*. The respondents were also asked if they had consulted a GP regarding the symptom. Moreover, items on self-reported lifestyle factors were included.

Participants’ unique, personal identification numbers in the CRS were used to obtain information on SES from Statistics Denmark, a governmental institution that tracks a variety of data on each Danish citizen [19].

### Statistical analysis

**Figure 1.**
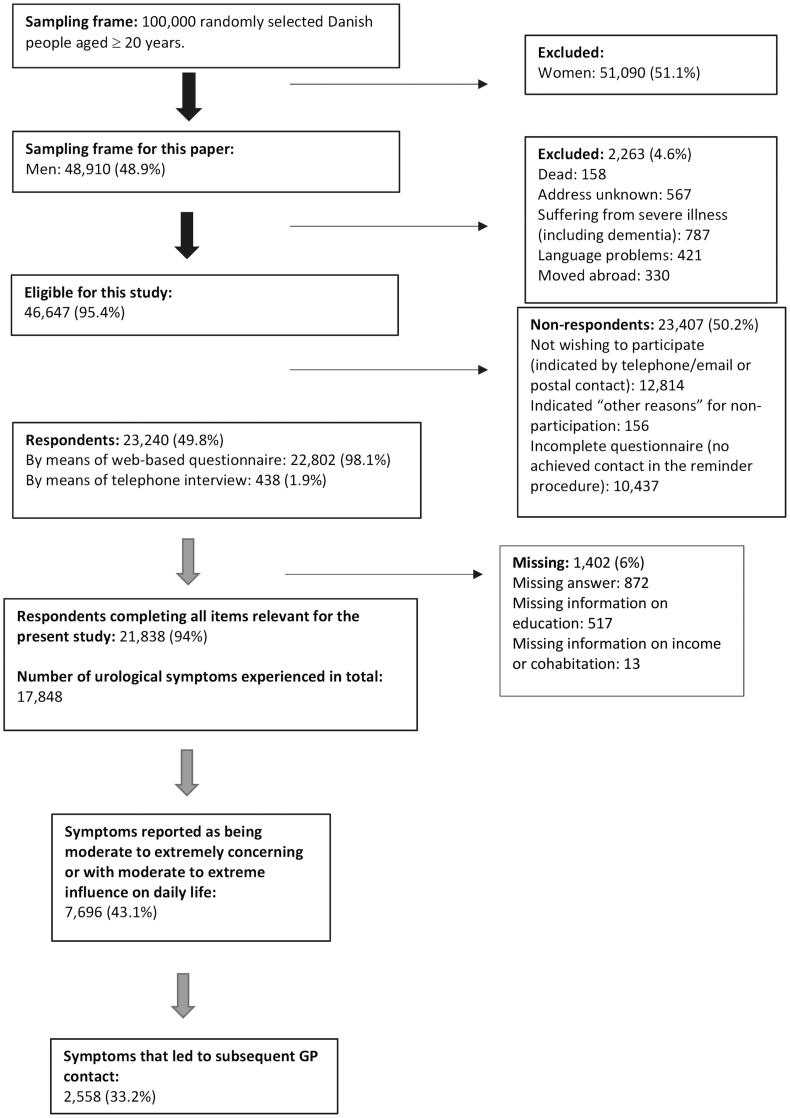
Flowchart of the study cohort (2012).

For this paper two datasets were constructed, one consisting of all reported LUTS and another consisting of a subgroup of LUTS reported to be ‘moderate to extremely concerning’ or with ‘moderate to extreme influence on daily activities’ (Figure 1). For practical purposes, the latter subgroup of symptoms will be referred to together as “bothersome symptoms” throughout this paper.

The proportions of men who experienced each LUTS were calculated as percentages of the total respondents. Logistic regression models were used to calculate the crude and adjusted odds ratios (ORs) for associations between (1) SES, lifestyle factors, and experience of each of the LUTS and (2) SES, lifestyle factors, and healthcare-seeking with each of the bothersome LUTS. Using a Wald test in the crude logistic analysis, any covariates with a p-value below 0.05 in the Wald test were adjusted for in the multivariable statistical analyses.

For all analyses, the three incontinence symptoms (urge incontinence, stress incontinence and incontinence without stress/urge) were merged into one group termed *incontinence,* leading to four symptom categories available for analysis: *difficult*y *in emptying the bladder*, *nocturia*, *frequent urination* and *incontinence*. Reasons for merging the incontinence symptoms included, among others, that no crucial differences in healthcare-seeking behavior were found regarding the three incontinence symptoms in a previous study [3]. The incontinence symptoms were included in the subgroup of bothersome symptoms according to the highest reported level of influence and concern. If any of the three incontinence symptoms was reported as being of moderate or greater concern/influence, *incontinence* as a group was included in the subgroup.

The covariates considered in the uni- and multivariable statistical analyses covered SES and lifestyle factors. The term SES will cover both socioeconomic and sociodemographic variables.

The respondents were divided into following age groups: 20–39, 40–59, 60–79 and ≥80 years. Income was defined as the average disposable income per person in a household and categorized according to quartiles: *low income* (first quartile), *middle income* (second and third quartile) and *high income* (fourth quartile). Education was categorized by the highest completed educational level (*<10 years, 10–12 years* and *>12 years*). Cohabitation status was categorized as *single* or *married/living together*. Labor market affiliation was categorized as follows: *working* (employees and students), *pensioners* (age-linked pension and early retirement), and *out of workforce* (unemployed, receiving social security or disability pension).

Lifestyle factors included BMI (*underweight: BMI <18.5, normal weight: BMI 18.5–24.9, overweight: BMI 25–29.5,* and *obese: BMI ≥30)*, smoking status (*never, former,* and *current*) and alcohol intake (*0, 1–7, 8–21,* and *22+ units per week*).

The symptom burden was measured as the number of urological symptoms and divided into three categories (*1, 2–3,* and *≥4*). For this variable, the three types of incontinence symptoms counted for one symptom each.

Missing data included respondents who did not answer all necessary questions and respondents for whom SES data was not obtainable in registers. Missing data was left out of the analyses.

All data analysis was conducted with StataIC 14© (StataCorp, College Station, TX, USA) and a significance level of *p* < 0.05 was used.

## Results

Of the 48,910 randomly selected Danish men invited to the survey, 46,647 men were found eligible for the study and 23,240 participated, resulting in a response rate of 49.8% (Figure 1). In total, 6% of all respondents were missing data, resulting in a total number of 21,838 respondents available for analysis (Figure 1).

Half the men reported experiencing nocturia within the four weeks previous to the survey, as shown in Table 1. Difficulty in emptying the bladder (14.5%), frequent urination (11.0%), and incontinence (6.5%) were less commonly reported but more often reported as bothersome (51.8%, 59.6% and 64.1%, respectively). GP contact regarding bothersome urological symptoms was more common for men reporting frequent urination (37.6%), incontinence (34.8%), and difficulty in emptying the bladder (38.5%), as compared to nocturia (28.8%).

**Table 1.** Characteristics of Danish men participating in the Danish Symptom Cohort (2012) with SES and lifestyle factors and stratified by symptom type.

	Respondents	Difficulty in emptying the bladder	Nocturia	Frequent urination	Incontinence^a^
	*n* (%)	*n* (%)	*n* (%)	*n* (%)	*n* (%)
Respondents					
Overall	21,838 (100.0%)	3159 (14.5%)	10,865 (49.8%)	2413 (11.0%)	1411 (6.5%)
Symptoms and GP contact					
GP contact with LUTS^b^		952 (30.1%)	1825 (16.8%)	682 (28.3%)	407 (28.8%)
Reporting LUTS as bothersome^c^		1637 (51.8%)	3716 (34.2%)	1438 (59.6%)	905 (64.1%)
GP contact with bothersome LUTS		631 (38.5%)	1071 (28.8%)	541 (37.6%)	315 (34.8%)
Age (years)					
<40	5082 (23.3%)	335 (6.6%)	1138 (22.4%)	382 (7.5%)	112 (2.2%)
40–59	8753 (40.1%)	992 (11.3%)	4105 (46.9%)	824 (9.4%)	354 (4.0%)
60–79	7423 (34.0%)	1670 (22.5%)	5172 (69.7%)	1086 (14.6%)	829 (11.2%)
>80	580 (2.7%)	162 (27.9%)	450 (77.6%)	121 (20.9%)	116 (20.0%)
Cohabitation status					
Single	4833 (22.1%)	643 (13.3%)	2098 (43.4%)	592 (12.2%)	316 (6.5%)
Married/living together	17,005 (77.9%)	2516 (14.8%)	8767 (51.6%)	1821 (10.7%)	1095 (6.4%)
Smoking status					
Never smoked	9205 (42.2%)	1061 (11.5%)	4155 (45.1%)	901 (9.8%)	452 (4.9%)
Previous smoker	7606 (34.8%)	1415 (18.6%)	4558 (59.9%)	956 (12.6%)	631 (8.3%)
Current smoker	5027 (23.0%)	683 (13.6%)	2152 (42.8%)	556 (11.1%)	328 (6.5%)
Alcohol intake (units/week)					
0	3619 (16.6%)	500 (13.8%)	1577 (43.6%)	533 (14.7%)	240 (6.6%)
1–7	10,143 (46.4%)	1391 (13.7%)	4835 (47.7%)	1091 (10.8%)	625 (6.2%)
8–21	6651 (30.5%)	1004 (15.1%)	3609 (54.3%)	651 (9.8%)	418 (6.3%)
22+	1425 (6.5%)	264 (18.5%)	844 (59.2%)	138 (9.7%)	128 (9.0%)
BMI (kg/m^2^)					
Underweight	107 (0.5%)	16 (15.0%)	33 (30.8%)	13 (12.1%)	9 (8.4%)
Normal	9066 (41.5%)	1179 (13.0%)	4213 (46.5%)	864 (9.5%)	510 (5.6%)
Overweight	9364 (42.9%)	1408 (15.0%)	4813 (51.4%)	1039 (11.1%)	612 (6.5%)
Obese	3301 (15.1%)	556 (16.8%)	1806 (54.7%)	497 (15.1%)	280 (8.5%)
Educational level					
<10 years	4054 (18.6%)	618 (15.2%)	2078 (51.3%)	572 (14.1%)	311 (7.7%)
10–12 years	10,748 (49.2%)	1506 (14.0%)	5196 (48.3%)	1241 (11.5%)	657 (6.1%)
>12 years	7036 (32.2%)	1035 (14.7%)	3591 (51.0%)	600 (8.5%)	443 (6.3%)
Household income					
Lowest quartile	3119 (14.3%)	418 (13.4%)	1297 (41.6%)	433 (13.9%)	241 (7.7%)
Middle quartile	11,211 (51.3%)	1648 (14.7%)	5488 (49.0%)	1302 (11.6%)	752 (6.7%)
Highest quartile	7508 (34.4%)	1093 (14.6%)	4080 (54.3%)	678 (9.0%)	418 (5.6%)
Labor market affiliation					
Working	15,372 (70.4%)	1692 (11.0%)	6562 (42.7%)	1361 (8.9%)	592 (3.9%)
Pension	4986 (22.8%)	1209 (24.2%)	3593 (72.1%)	824 (16.5%)	686 (13.8%)
Out of workforce	1480 (6.8%)	258 (17.4%)	710 (48.0%)	228 (15.4%)	133 (9.0%)

a The three incontinence symptoms (urge incontinence, stress incontinence and incontinence without stress/urge) were merged into one group termed *incontinence*.

b LUTS with any degree of concern or influence on daily activities.

^c^Bothersome meaning LUTS reported as either of moderate or greater concern or influence.

^d^The three incontinence symptoms (urge incontinence, stress incontinence and incontinence without stress/urge) were merged into one group termed *incontinence*.

Baseline characteristics differed to some extent among respondents and non-respondents. The median age among respondents was 53 years compared to 48 years among non-respondents. More respondents were single, had higher levels of education, higher likelihood of labor market affiliation, and higher income compared to non-respondents (data not shown).

Table 2 shows the ORs for reporting each LUTS with regard to SES and lifestyle. Overall, odds for reporting each LUTS significantly increased with increasing age, lack of labor market affiliation, and a BMI measuring in the obese range (Table 2).

**Table 2.** Estimated crude and adjusted odds ratios (ORs) with 95% confidence interval (CI) for reporting urological symptoms according to SES and lifestyle, stratified by symptom type, in 23,240 Danish men participating in the Danish Symptom Cohort (2012).

	Incontinence** N = 1411		Nocturia *N* = 10,865		Frequent urination *N* = 2413		Difficulty in emptying the bladder *N* = 3159	
	Crude OR (95% CI)	Adj. OR (95% CI)*	Crude OR (95% CI)	Adj. OR (95% CI)*	Crude OR (95% CI)	Adj. OR (95% CI)*	Crude OR (95% CI)	Adj. OR (95% CI)*
Age (years)								
< 40	1.0 (ref)	1.0 (ref)	1.0 (ref)	1.0 (ref)	1.0 (ref)	1.0 (ref)	1.0 (ref)	1.0 (ref)
40–59	**1.9 (1.5–2.3)**	**1.9 (1.5–2.3)**	**3.1 (2.8–3.3)**	**2.7 (2.5–3.0)**	**1.3 (1.1–1.5)**	**1.4 (1.2–1.6)**	**1.8 (1.6–2.1)**	**1.7 (1.5–2.0)**
60–79	**5.6 (4.6–6.8)**	**3.9 (3.1–5.0)**	**8.0 (7.3–8.6)**	**5.8 (5.2–6.5)**	**2.1 (1.9–2.4)**	**2.0 (1.7–2.3)**	**4.1 (3.6–4.7)**	**3.3 (2.9–3.8)**
> 80	**11.1 (8.4–14–6)**	**6.8 (4.9–9.4)**	**12.0 (9.8–14.7)**	**8.3 (6.6–10.5)**	**3.2 (2.6–4.1)**	**2.7 (2.1–3.6)**	**5.5 (4.4–6.8)**	**4.1 (3.2–5.3)**
Cohabitation status								
Single	1.0 (ref)	.	1.0 (ref)	1.0 (ref)	1.0 (ref)	1.0 (ref)	1.0 (ref)	1.0 (ref)
Married/living together	1.0 (0.9–1.1)	.	**1.4 (1.3–1.5)**	1.0 (1.0–1.1)	**0.9 (0.8–0.9)**	**0.9 (0.8–1.0)**	**1.1 (1.0–1.2)**	1.0 (0.9–1.1)
Smoking status								
Never smoked	1.0 (ref)	1.0 (ref)	1.0 (ref)	1.0 (ref)	1.0 (ref)	1.0 (ref)	1.0 (ref)	1.0 (ref)
Previous smoker	**1.8 (1.5–2.0)**	1.1 (1.0–1.3)	**1.8 (1.7–1.9)**	**1.1 (1.1–1.2)**	**1.3 (1.2–1.5)**	1.1 (0.9–1.2)	**1.8 (1.6–1.9)**	**1.2 (1.1–1.4)**
Current smoker	**1.4 (1.2–1.6)**	**1.2 (1.0–1.4)**	**0.9 (0.8–1.0)**	**0.8 (0.8–0.9)**	**1.1 (1.0–1.3)**	1.0 (0.9–1.1)	**1.2 (1.1–1.3)**	1.1 (1.0–1.2)
Alcohol intake								
0	1.1 (0.9–1.3)	1.1 (1.0–1.3)	**0.8 (0.9–0.9)**	1.0 (0.9–1.1)	1.4 (1.3–1.6)	**1.4 (1.2–1.5)**	1.0 (0.9–1.1)	1.1 (1.0–1.2)
1–7	1.0 (ref)	1.0 (ref)	1.0 (ref)	1.0 (ref)	1.0 (ref)	1.0 (ref)	1.0 (ref)	1.0 (ref)
8–21	1.0 (0.9–1.2)	0.9 (0.8–1.0)	**1.3 (1.2–1.4)**	**1.2 (1.1–1.2)**	**0.9 (0.8–1.0)**	**0.9 (0.8–1.0)**	**1.1 (1.0–1.2)**	1.0 (0.9–1.1)
22+	**1.5 (1.2–1.8)**	1.2 (1.0–1.5)	**1.6 (1.4–1.8)**	**1.3 (1.2–1.5)**	0.9 (0.7–1.1)	**0.8 (0.7–1.0)**	**1.4 (1.2–1.7)**	**1.2 (1.0–1.4)**
BMI (kg/m^2)								
Underweight	1.5 (0.8–3.1)	1.4 (0.7–2.9)	**0.5 (0.3–0.8)**	0.7 (0.4–1.0)	1.3 (0.7–2.4)	1.1 (0.6–2.0)	1.2 (0.7–2.0)	1.3 (0.7–2.2)
Normal	1.0 (ref)	1.0 (ref)	1.0 (ref)	1.0 (ref)	1.0 (ref)	1.0 (ref)	1.0 (ref)	1.0 (ref)
Overweight	**1.2 (1.0–1.3)**	1.1 (0.9–1.2)	**1.2 (1.1–1.3)**	1.0 (0.9–1.1)	**1.2 (1.1–1.3)**	**1.1 (1.0–1.2)**	**1.2 (1.1–1.3)**	1.0 (1.0–1.1)
Obese	**1.6 (1.3–1.8)**	**1.4 (1.2–1.6)**	**1.4 (1.3–1.5)**	**1.2 (1.1–1.3)**	**1.7 (1.5–1.9)**	**1.5 (1.3–1.7)**	**1.4 (1.2–1.5)**	**1.2 (1.0–1.3)**
Educational level								
< 10 years	1.0 (ref)	1.0 (ref)	1.0 (ref)	1.0 (ref)	1.0 (ref)	1.0 (ref)	1.0 (ref)	.
10–12 years	**0.8 (0.7–0.9)**	1.0 (0.9–1.2)	**0.9 (0.8–1.0)**	1.0 (1.0–1.1)	**0.8 (0.7–0.9)**	1.0 (0.9–1.1)	0.9 (0.8–1.0)	.
> 12 years	**0.8 (0.7–0.9)**	1.2 (1.0–1.4)	1.0 (0.9–1.1)	**1.1 (1.0–1.2)**	**0.6 (0.5–0.6)**	**0.8 (0.7–0.9)**	1.0 (0.9–1.1)	.
Household income								
Lowest quartile	1.0 (ref)	1.0 (ref)	1.0 (ref)	1.0 (ref)	1.0 (ref)	1.0 (ref)	1.0 (ref)	.
Middle quartile	**0.9 (0.7–1.0)**	0.9 (0.8–1.0)	**1.3 (1.2–1.5)**	**1.1 (1.0–1.2)**	**0.8 (0.7–0.9)**	0.9 (0.8–1.0)	1.1 (1.0–1.2)	.
Highest quartile	**0.7 (0.6–0.8)**	0.9 (0.7–1.1)	**1.7 (1.5–1.8)**	**1.3 (1.2–1.5)**	**0.6 (0.5–0.7)**	**0.8 (0.7–0.9)**	1.1 (1.0–1.2)	.
Labor market affiliation								
Working	1.0 (ref)	1.0 (ref)	1.0 (ref)	1.0 (ref)	1.0 (ref)	1.0 (ref)	1.0 (ref)	1.0 (ref)
Pension	**4.0 (3.6–4.5)**	**1.8 (1.5–2.1)**	**3.5 (3.2–3.7)**	**1.4 (1.2–1.5)**	**2.0 (1.9–2.2)**	**1.3 (1.1–1.5)**	**2.6 (2.4–2.8)**	**1.3 (1.1–1.4)**
Out of workforce	**2.5 (2.0–3.0)**	**2.0 (1.6–2.4)**	**1.2 (1.1–1.4)**	**1.2 (1.1–1.4)**	**1.9 (1.6–2.2)**	**1.4 (1.2–1.7)**	**1.7 (1.5–2.0)**	**1.5 (1.3–1.7)**

**Bold** indicating a *p* value below 0.05.

* Adjustments were made for all covariates that had a *p* value below 0.05 when tested using a Wald test.

** The three incontinence symptoms (urge incontinence, stress incontinence and incontinence without stress/urge) were merged into one group named *incontinence*.

**Figure 2.**
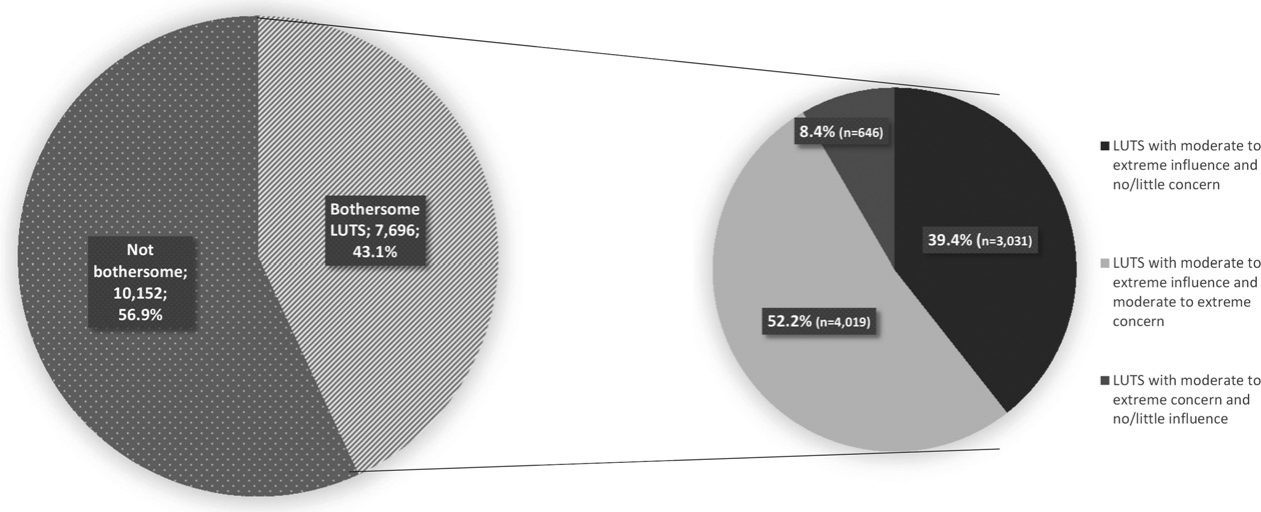
Distribution of all reported LUTS by bothersome and not bothersome, with bothersome LUTS further broken out to display: (1) The proportion of bothersome LUTS that are of moderate to extreme influence and no/little concern, (2) The proportion of bothersome LUTS that are of both moderate to extreme influence and moderate to extreme concern, and (3) The proportion of bothersome LUTS that are of moderate to extreme concern and with no/little influence on daily activities.

More than half of the bothersome symptom experiences (52.2%) were both moderate to extremely influencing on daily life and concerning, while few symptoms (8.4%) were reported as moderate to extremely concerning and with no/little influence on daily activity, as shown in Figure 2.

### SES

Table 3 depicts the associations between SES, symptom burden, lifestyle factors, and contact to GP regarding each of the bothersome LUTS.

**Table 3.** Estimated crude and adjusted odds ratios (ORs) with 95% confidence interval (CI) for GP contact regarding bothersome LUTS by symptom type with SES and lifestyle factors. Only bothersome symptoms were included in this analysis.

	Incontinence** (*N* = 905)			Nocturia (*N* = 3716)			Frequent urination (*N* = 1438)			Difficulty in emptying the bladder (*N* = 1637)		
	n	Crude OR (95% CI)	Adj. OR (95% CI)*	n	Crude OR (95% CI)	Adj. OR (95% CI)*	n	Crude OR (95% CI)	Adj. OR (95% CI)*	n	Crude OR (95% CI)	Adj. OR (95% CI)*
Age (years)												
< 40	68	1.0 (ref)	1.0 (ref)	332	1.0 (ref)	1.0 (ref)	188	1.0 (ref)	1.0 (ref)	158	1.0 (ref)	1.0 (ref)
40–59	233	1.3 (0.7–2.4)	1.1 (0.6–2.2)	1287	**2.1 (1.4–3.0)**	**1.9 (1.3–2.9)**	449	**1.9 (1.3–3.0)**	**1.9 (1.2–2.9)**	483	**2.4 (1.5–3.9)**	**2.2 (1.4–3.6)**
60–79	532	**2.3 (1.3–4.3)**	1.6 (0.8–3.3)	1899	**5.2 (3.6–7.5)**	**3.4 (2.3–5.1)**	727	**4.1 (2.7–6.1)**	**3.6 (2.3–5.7)**	906	**4.8 (3.1–7.6)**	**3.8 (2.3–6.3)**
> 80	72	**2.1 (1.0–4.5)**	1.3 (0.5–3.0)	198	**6.7 (4.3–10.6)**	**3.4 (2.1–5.7)**	74	**5.5 (3.1–10.0)**	**4.7 (2.4–9.2)**	30	**4.9 (2.7–8.9)**	**3.5 (1.8–6.8)**
Symptom burden												
1	65	1.0 (ref)	1.0 (ref)	1640	1.0 (ref)	1.0 (ref)	150	1.0 (ref)	1.0 (ref)	207	1.0 (ref)	1.0 (ref)
2–3	571	1.8 (1.0–3.5)	1.7 (0.9–3.3)	1835	**2.5 (2.2–3.0)**	**2.2 (1.9–2.6)**	1043	1.2 (0.8–1.7)	0.8 (0.5–1.2)	1192	**1.5 (1.1–2.1)**	1.2 (0.8–1.6)
4–6	269	**3.3 (1.7–6.4)**	**2.9 (1.5–5.8)**	241	**5.1 (3.9–6.8)**	**4.0 (3.0–5.3)**	245	**2.7 (1.8–4.1)**	**1.6 (1.0–2.6)**	238	**2.6 (1.7–3.8)**	**1.8 (1.2–2.7)**
Cohabitation status												
Single	202	1.0 (ref)	.	799	1.0 (ref)	.	378	1.0 (ref)	.	355	1.0 (ref)	.
Married/living together	703	0.9 (0.6–1.2)	.	2917	1.1 (0.9–1.3)	.	1060	1.2 (0.9–1.5)	.	1282	1.1 (0.9–1.4)	.
Smoking status												
Never smoked	280	1.0 (ref)	.	1205	1.0 (ref)	1.0 (ref)	496	1.0 (ref)	.	493	1.0 (ref)	.
Previous smoker	404	1.2 (0.9–1.6)	.	1706	**1.2 (1.0–1.4)**	0.9 (0.8–1.1)	583	**1.3 (1.0–1.7)**	.	758	1.1 (0.9–1.4)	.
Current smoker	221	0.6–1.3)	.	805	**0.8 (0.6–1.0)**	**0.7 (0.6–0.9)**	359	1.1 (0.8–1.5)	.	386	0.9 (0.7–1.2)	.
Alcohol intake												
0	160	1.1 (0.8–1.7)	.	690	1.0 (0.8–1.2)	.	333	0.8 (0.6–1.0)	.	287	1.1 (0.9–1.5)	.
1–7	403	1.0 (ref)	.	1600	1.0 (ref)	.	654	1.0 (ref)	.	719	1.0 (ref)	.
8–21	255	0.7 (0.5–1.0)	.	1123	0.9 (0.8–1.1)	.	357	0.9 (0.7–1.2)	.	491	0.9 (0.7–1.1)	.
22+	87	0.7 (0.4–1.1)	.	303	**0.7 (0.5–1.0)**	.	94	0.7 (0.4–1.1)	.	140	0.7 (0.5–1.0)	.
BMI (kg/m^2)												
Underweight	6	1.7 (0.3–8.8)	.	17	1.4 (0.5–3.9)	.	10	1.1 (0.3–4.0)	.	10	1.2 (0.3–4.4)	.
Normal	305	1.0 (ref)	.	1249	1.0 (ref)	.	486	1.0 (ref)	.	566	1.0 (ref)	.
Overweight	404	1.0 (0.7–1.4)	.	1673	1.1 (0.9–1.3)	.	641	1.1 (0.8–1.4)	.	740	1.3 (1.0–1.6)	.
Obese	190	0.7 (0.5–1.0)	.	777	1.1 (0.9–1.4)	.	301	0.9 (0.7–1.2)	.	321	1.2 (0.9–1.5)	.
Educational level												
<10 years	204	1.0 (ref)	.	858	1.0 (ref)	.	363	1.0 (ref)	.	357	1.0 (ref)	1.0 (ref)
10–12 years	430	0.9 (0.6–1.2)	.	1884	0.9 (0.7–1.0)	.	753	0.9 (0.7–1.1)	.	811	**0.7 (0.5–0.9)**	**0.8 (0.6–1.0)**
>12 years	271	0.9 (0.6–1.4)	.	974	1.0 (0.8–1.2)	.	322	1.1 (0.8–1.5)	.	469	0.8 (0.6–1.0)	0.8 (0.6–1.1)
Household income												
Lowest quartile	151	1.0 (ref)	1.0 (ref)	557	1.0 (ref)	.	276	1.0 (ref)	.	243	1.0 (ref)	.
Middle quartile	500	0.7 (0.5–1.1)	0.8 (0.5–1.2)	2040	0.8 (0.7–1.0)	.	765	0.9 (0.7–1.2)	.	872	0.9 (0.7–1.2)	.
Highest quartile	254	**0.6 (0.4–0.9)**	0.7 (0.4–1.1)	1119	**0.8 (0.6–1.0)**	.	397	0.8 (0.6–1.2)	.	522	0.9 (0.7–1.3)	.
Labor market affiliation												
Working	359	1.0 (ref)	1.0 (ref)	1922	1.0 (ref)	1.0 (ref)	711	1.0 (ref)	1.0 (ref)	782	1.0 (ref)	1.0 (ref)
Pension	450	**2.2 (1.6–2.9)**	1.5 (1.0–2.4)	1453	**2.7 (2.3–3.1)**	**1.5 (1.2–1.9)**	557	**2.2 (1.7–2.8)**	1.1 (0.8–1.5)	694	**2.1 (1.7–2.6)**	1.2 (0.9–1.6)
Out of workforce	96	**2.1 (1.3–3.3)**	**1.8 (1.1–3.0)**	341	**1.5 (1.2–2.0)**	**1.3 (1.0–1.8)**	170	1.2 (0.9–1.8)	1.1 (0.8–1.6)	161	1.3 (0.9–1.9)	1.1 (0.8–1.6)

**Bold** indicates a *p* value below 0.05.

*Adjustments were made for all covariates that had a *p* value below 0.05 when tested using a Wald test.

**The three incontinence symptoms (urge incontinence, stress incontinence and incontinence without stress/urge) were merged into one group named *incontinence*.

Higher age was significantly associated with higher odds for GP contact regarding bothersome nocturia, difficulty in emptying the bladder, and frequent urination (Table 3). Being out of the workforce significantly increased odds for GP contact regarding bothersome incontinence (OR = 1.8, 95% CI = 1.1–3.0) and nocturia (OR = 1.3, 95% CI = 1.0–1.8) compared to individuals who were in the workforce, as shown in Table 3.

## Symptom burden

Overall, reporting four to six symptoms significantly increased the odds for GP contact as compared to reporting one symptom (Table 3).

## Lifestyle factors

In general, no significant associations were shown between lifestyle factors and GP contact for any of the four bothersome LUTS, except for nocturia. For bothersome nocturia, current smokers had significant lower odds for GP contact (OR = 0.7, 95% CI = 0.6–0.9) compared to non-smokers (Table 3).

## Discussion

### Summary of principal findings

This large population-based study found that urological symptoms were often reported as bothersome but only one-third of men reporting bothersome LUTS contacted their GP. Increasing age, obesity, and having no affiliation with the labor market all significantly increased the odds for reporting LUTS. Factors associated with GP contact regarding bothersome LUTS were increased age and symptom burden. The decision to contact a GP was, however, not found to be influenced by lifestyle or SES.

### Strengths and limitations

The large randomly selected study population is a major strength of this study. To our knowledge, this is the largest population-based study covering this topic. The response rate (49.8%) corresponds to rates reported in other population-based studies [12,20].

Using a web-based questionnaire may have precluded individuals from participating in the study, potentially limiting the participation of, for instance, the elderly or individuals without access to the internet. This potential selection bias was sought to be minimized by offering the option of a telephone survey [21]. Selection bias is difficult to exclude completely and in the present study some differences of SES in respondents versus non-respondents were found. This may mask possible inequalities across SES, leading to an imprecise estimate of symptom experiences and healthcare-seeking behavior among or between socioeconomic groups.

Urological symptom experiences were self-reported for which reason recall bias cannot be eliminated. However, this was sought to be minimized by specifying a limited recall time frame of four weeks [22]. In addition, lifestyle factors were also self-reported, hence the possibility of information bias cannot be eliminated [23,24]. The literature indicates that LUTS are associated with patient experiences of shame and embarrassment [8,9], and consequently the prevalence of LUTS may be underreported. This issue may have been minimized by our use of a web-based questionnaire, which could increase respondents’ perceptions of privacy and thereby increase the reliability of answers regarding sensitive items.

Within the present study, a standardized score like the International Prostate Symptom Score (IPSS) was not used when questioning respondents about LUTS. Additionally, four different urological symptoms were included instead of only one, or several more, as in other studies [4,12,25,26]. This may complicate comparison of our results to different studies. Stratification performed by symptom type in the data may, however, provide opportunities for comparison with other studies that have examined single symptoms.

Instead of focusing on healthcare-seeking behavior with LUTS in general, this paper addresses a group of urological symptoms reported to be at least moderately concerning or influencing on daily activities. The specific subgroup of bothersome LUTS was chosen because LUTS are often of benign origin [2] and mild cases often do not necessarily require GP treatment. The label ‘bothersome’ was chosen for practical purposes and hence, the term was not validated and is the author groups interpretation.

When examining the subgroup of LUTS that were either concerning or influencing, symptoms reported as moderate to extremely influencing but ‘not at all’ or ‘slightly’ concerning or vice versa were also included. This may influence the results regarding healthcare-seeking behavior since symptoms that are both of extreme concern and extreme influence might be more likely to need GP attention than symptoms of moderate concern but with no influence on daily activities, or no concern and only moderate influence on daily activities. The results showed that few LUTS reached the level of moderate concern without influence on daily activities also being reported, whereas the opposite scenario (no or only slight concern, but moderate influence on daily activities) amounted to almost 40%. This may indicate that LUTS often interfere with daily activities even when the symptoms are not concerning, whereas concerning LUTS often also influence daily activities. However, a detailed discussion of this is beyond the scope of this paper.

### Discussion of findings and existing literature

Several studies have examined healthcare-seeking behavior in men with LUTS [12,25], but only a few have explored patient contact with GPs when LUTS are reported as bothersome. Boyle et al found in a population-based study that 40.9–77.5% of men aged 40–79 years consulted a doctor with bothersome incontinence [25] compared to 34.8% in the present study. The difference might be due to differences in study design, including age groups, definition of bothersome symptoms, and recall-period.

Increasing age was found to be the most substantial risk factor for reporting LUTS regardless of the symptom type in the present study. This supports findings in previous studies [7,26,27]. In the present study, increasing age was the strongest determinant of GP contact regarding bothersome LUTS, aside from incontinence. This finding is consistent with existing literature [12,25] regarding healthcare-seeking behavior with LUTS regardless of level of concern or influence on daily activities.

Obesity was found to be associated with LUTS, which was also found in previous studies [7,26,27]. Seim et al showed that both men with a BMI between 30–34 and 35–39 had increased odds for LUTS compared to men with BMI below 25 [26]. In a review by Parsons et al., the findings regarding associations between BMI and odds of reporting LUTS were ambiguous [6]. Some studies found increased odds for reporting LUTS with increasing BMI, while two studies did not find any association [6]. This could be due to the different categorizations of BMI. Although a BMI of greater than or equal to 30 was found to increase the likelihood of reporting LUTS in the present study, no associations were found between BMI ≥30 and contact with GP regarding LUTS.

Studies examining lifestyle factors and GP contact regarding LUTS are limited. Several studies, however, have examined associations between lifestyle factors and GP contact regarding different symptoms [17,28,29], and the various results indicate that lifestyle factors affect the healthcare-seeking behavior differently according to different symptoms.

Very few studies have examined the associations between SES and LUTS [7]. Wang et al’s population-based study on the Chinese population found that higher educational levels decreased the risk of reporting urological storage and voiding symptoms [7]. However, that study’s different methodology regarding education level and number of symptoms make comparison difficult. No other studies have analyzed the association of LUTS and SES. However, a systematic review by Yousaf et el found that both men with low income and unmarried men were less likely to seek medical treatment or advice regarding any symptom [14]. This was not found in the present study regarding bothersome LUTS.

### Meaning of the study

This study showed that even though LUTS are often concerning and influence the daily activities of Danish men, only one-third of such symptoms are discussed with a GP. In this study, advanced age and symptom burden appear to be the only significant contributing factors for consulting a GP regarding bothersome LUTS. These findings could indicate that regardless of SES and lifestyle Danish men may lack knowledge about treatments and advice that GPs can provide to reduce the bothersome LUTS. These findings may be valuable to GPs and other healthcare workers in order to detect and treat men suffering from bothersome LUTS. The knowledge may also be beneficial for public health initiatives to destigmatize LUTS among men in Denmark, e.g. information campaigns on management and treatment options for LUTS or encouraging men to consult a physician when bothered by urological symptom(s). Future research should examine possible barriers to healthcare-seeking among men experiencing bothersome LUTS, as this knowledge may help to provide better care for these individuals.
